# Management and outcomes of acute ST-segment-elevation myocardial infarction at a tertiary-care hospital in Sri Lanka: an observational study

**DOI:** 10.1186/1471-2261-15-1

**Published:** 2015-01-15

**Authors:** Ruwanthi Bandara, Arjuna Medagama, Ruwan Munasinghe, Nandana Dinamithra, Amila Subasinghe, Jayantha Herath, Mahesh Ratnayake, Buddhini Imbulpitiya, Ameena Sulaiman

**Affiliations:** Professorial Medical Unit, Teaching Hospital Peradeniya, Peradeniya, Sri Lanka; Department of Medicine, Faculty of Medicine, University of Peradeniya, Peradeniya, Sri Lanka

**Keywords:** ST-segment-elevation myocardial infarction, STEMI, Thrombolysis, Door-to-needle time, Survival, 1-year mortality, Sri Lanka

## Abstract

**Background:**

Sri Lanka is a developing country with a high rate of cardiovascular mortality. It is still largely dependent on thrombolysis for primary management of acute myocardial infarction. The aim of this study was to present current data on the presentation, management, and outcomes of acute ST-segment-elevation myocardial infarction (STEMI) at a tertiary-care hospital in Sri Lanka.

**Methods:**

Eighty-one patients with acute STEMI presenting to a teaching hospital in Peradeniya, Sri Lanka, were included in this observational study.

**Results:**

Median interval between symptom onset and hospital presentation was 60 min (mean 212 min). Thrombolysis was performed in 73% of patients. The most common single reason for not performing thrombolysis was delayed presentation. Median door-to-needle time was 64 min (mean, 98 min). Only 16.9% of patients received thrombolysis within 30 min, and none underwent primary PCI. Over 98% of patients received aspirin, clopidogrel, and a statin on admission. Intravenous and oral beta blockers were rarely used. Follow-up data were available for 93.8% of patients at 1 year. One-year mortality rate was 12.3%. Coronary intervention was performed in only 7.3% of patients post infarction.

**Conclusion:**

Late presentation to hospital remains a critical factor in thrombolysis of STEMI patients in Sri Lanka. Thrombolysis was not performed within 30 min of admission in the majority of patients. First-contact physicians should receive further training on effective thrombolysis, and there is an urgent need to explore the ways in which PCI and post-infarction interventions can be incorporated into treatment protocols.

## Background

Sri Lanka is a developing country with a population of 21 million and a rapidly increasing burden of non-communicable diseases.

In 2005, the prevalences of hypertension, diabetes, and dysglycemia were 20%, 11%, and 20%, respectively [1]. Mortality from cardiovascular disease is recognized to be one of the highest worldwide [2]. Ischemic heart disease (IHD) is the leading cause of death in Sri Lanka and accounts for 27.6 deaths per 100,000 people. Data from 2004 to 2012 show a steady increase in hospital mortality from IHD; in 2012 it accounted for 14.4% all hospital deaths [3]. This high mortality remains unexplained but may be attributable in part to the clustering of modifiable and non-modifiable cardiovascular risk factors.

Acute coronary syndrome (ACS) encompasses a spectrum of coronary artery diseases, including unstable angina, ST-segment-elevation myocardial infarction (STEMI), and non-STEMI.

There has recently been a worldwide transition in the acute management of ACS. Primary percutaneous coronary intervention (PCI) is steadily being introduced in most parts of the developed world and middle-income countries. However, in Sri Lanka, ACS is still managed primarily medically and, in most instances, in non-specialized general medical units. Rates of secondary prevention of cardiovascular disease are low in middle-income countries like Sri Lanka, but offer substantial oppurtunties for further development [4].

Clinical trials have provided clinicians with many evidence-based interventions and medications. These are augmented by observational studies, which provide useful information about patients hospitalized for acute coronary events [5]. Observational studies have revealed differences and shortcomings in management practices among countries as well as within different regions of the same country [6, 7]. The development of patient registries is an important step toward increased awareness of cardiovascular disease and establishment of appropriate management strategies. Although developed countries already have such registries, these need to be established in developing countries so that current preventive and management strategies can be audited.

At present, there is little information on the management and outcomes of acute myocardial infarction (AMI) [8] in Sri Lanka. The aim of this study was to examine current data on the presentation, management, and outcomes of acute STEMI at a tertiary-care hospital in Sri Lanka.

## Methods

This prospective observational study was carried out at the Professorial Medical Unit of Peradeniya Teaching Hospital, Sri Lanka, over a 6-month period beginning November 2011. This is a busy state-run tertiary-care institution with a nonselective intake of acute patients.

To be eligible for enrollment, patients had to present within 24 h of onset of symptoms likely to be of myocardial origin. In addition, they had to have electrocardiographic (ECG) changes fulfilling current ECG criteria in the diagnosis of acute STEMI or new-onset left bundle-branch block, and an increase in a biochemical marker of myocardial necrosis where results were available. All patients presenting with chest pain within the preceding 24 h who fulfilled ECG criteria for acute STEMI were included in the study. A diagnosis of STEMI was made by a senior house officer or registrar on admission and later confirmed by a consultant physician. Troponin level is not checked at most government hospitals; when required, the test is performed in a private laboratory at the patient’s expense.

Cardiac troponin levels were therefore not routinely available to us and were requested only in instances where diagnosis was uncertain. Assessment with history, physical examination, and ECG was performed for every patient presenting with chest pain, and those patients fulfilling the criteria for STEMI were included in the present study. Patients provided written informed consent prior to collection of management data, which were extracted from interviews conducted by the authors and from hospital records.

Following hospital discharge, patients were allowed to be followed up at their usual clinics. One year from the onset of STEMI, patients were invited for another interview with the authors. At this visit, patients’ current symptoms and examination findings were recorded and data from clinic records regarding management during the previous year was obtained. An echocardiogram was performed to assess left ventricular function and to detect new onset of regional wall-motion abnormalities and other structural abnormalities.

The study did not alter the standard care given to the patients. Ethical clearance for this study was obtained from the ethics review committee of the faculty of medicine at University of Peradeniya, Sri Lanka (2011/EC/22).

Binary logistic regression was used to assess the association of mortality at 1 year with history of smoking, hypertension, dyslipidemia, time taken for presentation, door-to-needle time, ejection fraction, and hypotension during the index admission and the mode (direct vs. redirected) of admission to the hospital. A P value less than 0.05 was considered to be statistically significant.

## Results

There were 81 admissions with confirmed STEMI during the study period. There were 21 (26%) females and 60 (74%) males and the mean age was 61.7 (SD 10.7) years. The majority of patients (59.5%) belonged to the age group of 50–69 years, and a further 20% were from the 70–79 year group.

Seventeen (21%) were current smokers, 47 (58%) had smoked at some time during their lives and 34 (42%) patients had never smoked. None of the females had ever smoked. Seven patients (8.6%) had previous history of ischaemic heart disease. Twenty two (27%) had a history of hypertension, 25 (30.8%) diabetes mellitus and 6 (7%) isolated dyslipidaemia. Socio-demographic profile, co-morbidities and risk factors are gven in Table 1.

**Table 1:** **Patients’ socio-demographic variables and risk factors**

**Gender**		No	(%)
	Males	21	26
	Females	60	74
**Age**		**Mean (years)**	**SD (years)**
	Males	59.38	10.62
	Females	70.70	8.67
**BMI (kg/m** ^**2**^ **)**		**Mean**	**SD**
	Males	25.12	6.64
	Females	23.59	3.73
**Ethnicity**		**No**	**(%)**
	Sinhalese	59	73
	Muslim	14	17
	Tamil	08	10
**Comorbidities and risk factors**		**Frequency**	**(%)**
	Hypertension	22	27
	Diabetes mellitus	25	30.8
	Dyslipidemia	6	7
	Ischemic Heart Disease	7	8.6
	Smoking	Males (n)	Females (n)
	Current	17	0
	Ex	30	0
	Never	34	0

BMI, body mass index.

There were 45 (58%) direct admissions to Teaching Hospital Peradeniya (THP), 23 (27%) were referred from the regional hospitals and 11 (12.5%) referred by the general practitioner (GP).

Time to presentation by direct or redirected admission is presented in Table 2. Of the patients presenting directly to our facility, median time from symptom onset to admission was 60 min, the mean was 212 min. Patients who had been seen at a peripheral hospital or by a general practitioner took longer to present (median, 75 min; mean, 281 min). The difference did not reach statistical significance (P = 0.45).

**Table 2:** **Comparison of time to presentation between direct and redirected admission**

	Time taken for admission to tertiary care center (minutes)	
	Median	Mean
**Direct admission (n = 45)**	60	212
**Re-directed admissions (n = 36)**	75	281

P = 0.45.

Seventy seven patients (95%) presented with chest pain and four patients had symptoms other than chest pain. Forty three patients (53%) had anterior STEMI or new-onset left bundle-branch block, 32 (40%) had inferior STEMI, and six (7%) had lateral STEMI. Of the 81 patients studied, 10 (12.3%) were not eligible for thrombolysis because of late presentation; thus, 71 patients (87%) were eligible for thrombolytic therapy, and 59 (72.8%) underwent thrombolysis with streptokinase.

Twelve patients were therefore eligible on the basis of arrival time, but three of these (3.7%) had absolute or relative contraindications to thrombolysis and nine (11.1%) eligible patients did not undergo thrombolysis for unknown reasons. The 12 patients who were eligible for, but did not undergo, thrombolysis and those who presented late were treated with either low-molecular-weight heparin or unfractionated heparin.

Median door-to-needle time was 64 min (mean, 98 min). Ten patients (16.9%) underwent thrombolysis within 30 min, 28 (47.4%) within 60 min and 39 (66%) within 90 min. Twenty (33.9%) underwent thrombolysis more than 90 min after arriving at the hospital. Time to thrombolytic therapy is shown in Table 3.

**Table 3:** **Door-to-needle times of patients receiving streptokinase**

	Number	Cumulative percentage (%)
**≤30 minutes**	10	16.9
**>30 ≤ 60 minutes**	18	47.4
**>60 ≤ 90 minutes**	11	66
**>90 minutes**	20	100

Of the 81 patients presenting to our facility, 99% received aspirin, 97% received clopidogrel, and 95% received a statin on admission. Oxygen was given to 79%, analgesics to 61%, and oral beta blockers to 35%. Only 16% of patients presenting to either a peripheral hospital or a general practitioner received aspirin.

During hospitalization, patients were administered aspirin (91%), clopidogrel (95%), statin (91%), angiotensin-converting-enzyme inhibitor (ACEI) or angiotensin-receptor blocker (ARB) (95%), and nitrates (60%). At discharge, over 88% received a combination of aspirin, clopidogrel, statin, and ACEI or ARB. At discharge, only 59% of patients were prescribed nitrates and only 34% were prescribed beta blockers.

Following thrombolysis, four patients (5%) were managed in the intensive care unit, 38 (47%) in the high-dependency unit, and remainder in the general medical unit. One patient developed gastrointestinal bleeding following thrombolysis.

Mean duration of hospital stay following acute STEMI was 5.5 days. There was no difference in duration of stay between patients who experienced anterior STEMI and those who experienced STEMI located elsewhere. During hospitalization, one patient developed bleeding while on low-molecular-weight heparin, four had reversible arrhythmias, seven developed heart failure, four had heart failure with reversible cardiogenic shock, and two developed pneumonia.

A pre-discharge echocardiogram was performed in 72 patients (88%), of whom 10 (12.3%) had an ejection fraction < 40%. Mean ejection fraction of the study population during the index admission was 48.4% (range, 20–68%).

Follow-up data were available for 93.8% of patients. At the end of 1 year, 66 (81.5%) of the 81 patients with STEMI were reassessed, five (6.1%) were lost to follow-up, and 10 (12.3%) had died. Thirty-seven patients (45.6%) had been referred to a specialized cardiology unit, 22 (27%) underwent exercise ECG testing, 10 (12.3%) underwent coronary angiography, five (6%) underwent percutaneous transluminal coronary angiography, and two (2.3%) underwent coronary artery bypass grafting. Six patients had been readmitted for cardiac-related problems, four for heart failure associated with ACS, and two for ACS. Outcomes of patients at 1-year is tabulated in Table 4.

**Table 4:** **One-year outcomes**

	Number	Percentage (%)
**1 year follow up**	66	81.5
**Lost to follow up**	5	6
**Deceased at 1 year**	10	12.3
**Referral done to specialized cardiology center**	37	45.6
**Underwent Exercise ECG**	22	27
**Underwent Coronary angiogram**	10	12.3
**Underwent Revascularization**	7	7.3
**Re-admitted with Coronary events**	6	7.4

ECG, electrocardiogram.

At the end of 1 year, six patients (7%) were still smoking; mean systolic and diastolic blood pressures were 128 mmHg and 79 mmHg, respectively; and medications being used were aspirin (83%), clopidogrel (82%), statin (90%), and ACEI or ARB (85%).

Echocardiography at 1-year follow-up revealed new-onset regional wall-motion abnormalities in six patients (9%), of whom five demonstrated new-onset left ventricular dysfunction (ejection fraction <40%). Mean ejection fraction at 1 year was 49%.

Of the ten patients who died, four had anterior STEMI and six had inferior myocardial infarction during the index admission. The immediate cause of death is not known. Thrombolysis was not received by one patient. There was no significant difference in mean door-to-needle time between patients who died within the first year (129 min) and those who were alive at 1-year follow-up (92 min) (*P* = 0.3). Mean time to hospital presentation was 61.8 min in those who died and 247 min in those who survived. Mean ejection fraction at the index admission did not differ significantly between non-surviving patients (49%) and surviving patients (49%). Only one patient had post-infarction heart failure during the index admission. Two patients were referred to a cardiologist. The deceased patients had received aspirin, clopidogrel, and a statin at admission and at discharge. The presence of diabetes mellitus (*P* = 0.01) and previous IHD (*P* = 0.05) were associated with death at 1 year.

No significant association was found between death at 1 year and history of smoking, hypertension, dyslipidaemia, time taken for presentation, door to needle time, ejection fraction and hypotension during the index admission and the mode of admission to hospital.

## Discussion

STEMI was diagnosed with reasonable accuracy in our study by clinical and ECG criteria. Troponin levels were obtained in a minority of patients because of the unavailability of the test in the state sector and the high cost in private-sector laboratories.

Despite coronary vascular disease being the largest cause of mortality among Sri Lankan adults, no study has described the presentation, management, and outcomes of STEMI; likely because of lack of resources and funding. Peradeniya Teaching Hospital is one of the two largest tertiary-care institutions in the area, and many smaller hospitals redirect patients with ACS to this unit; the sample can thus be considered representative. Percutaneous Coronary Interventions (PCI) or CABG is not performed at this hospital and specialized cardiology services are available only at the Teaching Hospital Kandy situated 4 km away. At the time of the study Primary PCI was not routinely performed at this instituttion either.

The prevalences of smoking (58%) and diabetes (30.8%) in this study are comparable with data from regional and global studies [5, 9]. The prevalences of hypertension, dyslipidemia, and preexisting coronary heart disease (Table 1) were considerably lower than in other studies. Mohanan et al. reported the prevalence of hypertension and previous IHD among patients in Kerala, India, presenting with ACS to be 48% and 14% [9]; the Access study investigators reported higher prevalences of 65% and 26%, respectively [5]. The prevalences of hypertension and diabetes among adult Sri Lankans are 23% and 10.8%, respectively [10, 11], but they seem to be underreported in the current study. In Sri Lanka, the first recognition of risk factors for cardiovascular disease is often at presentation for STEMI and may explain the low prevalence of risk factors reported in the current study.

Late presentation; contraindications; and other factors that are undocumented and therefore unknown rendered 27% of patients ineligible for thrombolysis following STEMI. Better training of staff can facilitate recognition of the need for thrombolysis at presentation. Better record keeping is also essential.

Delayed presentation resulting in ineligibility for thrombolysis was mainly pre-hospital, and similar delays have been observed in other studies carried out in Sri Lanka. Constantine et al. reported median pre-hospital delays of 130 min in 1997 and 720 min in 1999, and the delay was longer in patients who sought pre-hospitalization medical advice than in those who presented directly [12, 13]. In our study, median delay was 60 min (mean, 212 min) in patients presenting directly to our center and 75 min (mean 281 min) in those presenting through another hospital or general practitioner, a trend similar to those reported previously [13]. However it did not reach statistical significance. The longer mean than median time to presentation is the result of several patients presenting more than 12 hours following onset of pain. Mohanan et al. recently reported time from ACS symptom onset to ER presentation of over 6 hours in India [9].

While time to presentation has greatly improved, there is still considerable delay at peripheral hospitals and clinics, and while we did not identify an association between time to presentation and 1-year mortality, this could have been because of our relatively small sample size.

In the present study, 83% of eligible patients underwent thrombolysis, a result consistent with that of Rajapakse et al., who reported that thrombolysis was performed in 84.6% of a cohort of AMI patients at another tertiary-care institution in Sri Lanka, the National Hospital Colombo, in 2008 [8]. Both figures show a tremendous improvement from the 17% reported in 1999 [13]. However a large proportion of patients with STEMI (27%) did not receive thrombolysis or PCI in our study. Access investigators who studied the outcomes of ACS patients in the developing countries of Latin America, the Middle East, and Africa reported that 39% of STEMI patients did not receive thrombolysis or PCI [5]. However, in this multi-national survey, which included 11,731 patients, the overall rates for angiography and PCI were 58 and 35%, respectively.

Our data suggest that time delay to presentation to a tertiary-care center is a critical factor that reduced patients’ eligibility for thrombolysis. Reasons for this delay are likely 1) the absence of organized emergency medical transport [9] and cardiac response teams, requiring patients to provide their own transportation to the hospital; and 2) lack of patient awareness regarding ACS.

The benefits of early thrombolytic therapy are well established. Mean absolute reduction in mortality per hour of delay is 1.6 (standard deviation, 0.6) per 1,000 patients [12]. Median door-to-needle time in our study was 64 min; the mean of 96 min was due to late thrombolysis in some patients because of difficulty making a definitive diagnosis of STEMI. Guidelines recommend a door-to-needle time of less than 30 min. This was achieved in only 16.9% of our patients, but thrombolysis was performed within 60 min of admission in 47% and within 90 min of admission in 66%. In a previous study, a median door-to-needle time of 70 min was reported [12]. In Malaysia the median door-to-needle time was 48–54 min in a prospective study in which medical and emergency department doctors administered thrombolysis [14]. Mohanan et al. reported that less than one-third of patients undergoing thrombolysis had door-to-needle times of more than 30 min in India [9]. Door-to-needle time is a critical factor in the management of STEMI and first-contact doctors at all hospitals should be educated in early diagnosis and immediate thrombolysis.

More than 95% of STEMI patients received aspirin, clopidogrel, and a statin on presentation to our unit. However, a considerable proportion (84%) of STEMI patients who presented initially to another hospital or a general practitioner did not receive aspirin prior to being referred for thrombolysis. In 2010, Rajapakse et al. reported that aspirin and clopidogrel loading was performed in only 69% and 61% of patients, respectively [8]. Our use of aspirin and clopidogrel on admission seems to be satisfactory and compares well with other studies of the region [9] and of the developing world in general [5]. Continued therapy with aspirin, clopidogrel, and statin medication was observed in more than 88% of participants during hospitalization and on discharge. Aspirin was the medication most likely to be withheld, mostly for reasons of epigastric pain or presumed gastrointestinal hemorrhage. An ACEI or ARB was administered to over 90% of patients during hospitalization and to over 88% on discharge. At the end of 1 year, the use of the above medications remained over 82%. Access investigators who studied over 11,000 patients with ACS in the developing world reported the use of ACEIs to be 68% [5]. The World Health Organization considers the use of aspirin, statin and blood pressure-lowering agents post discharge in ACS patients to be cost-effective strategies and are considered *best buys* [15]. Nitrates were prescribed to more than 59% of patients during hospitalization and on discharge.

Intravenous beta blockers were not used in the acute management of STEMI in the present study or in previous Sri Lankan studies. However 35% of patients received oral beta blockers during the initial assessment. Intravenous beta blockers followed by oral beta blockers should be administered immediately, in the absence of contraindications, to all patients with STEMI [16]. Underutilization of intravenous beta blockers persists and should be analyzed in future studies. Oral beta blockers were prescribed at discharge to 34% of our study patients, compared with 78% in other developing countries [5].

Eighteen (22%) of our patients had complications during the post-infarction period, the majority of which were from cardiac causes. Only one patient developed a major gastrointestinal hemorrhage following thrombolytic therapy. Seventy six (85%) of our patients had pre-discharge echocardiography performed and 14% had evidence of left ventricular dysfunction, defined as an ejection fraction <40%, at the time of discharge. Rajapakse et al. also reported a complication rate of 25% in their ACS patients [8]. Both studies show an improvement from the previously reported incidence of 37% [13] that may be due to the higher adherence to guidelines and wider use of thrombolysis. There were no in-hospital deaths during the index admission in the present study, which probably reflects our small sample size. Mohanan et al. reported an in-hospital mortality of 4.3–8.6% among various cardiac registries globally [9].

This study examined 1-year survival of post-STEMI patients in Sri Lanka. Ten of the initial 81 STEMI patients were deceased at 1-year follow-up. The 1-year mortality of 12.3% in our study was slightly greater than the observed value of 8–10% in the major thrombolysis trials [17]. Similarly, an all-cause death rate of 7.3% at 1 year was observed by the Access investigators [5]. The higher 1-year mortality observed in the present study is probably the result of late presentation at the index admission, underutilization of thrombolysis, a longer-than-recommended door-to-needle time, and a very low rate of coronary intervention in the post-infarction period. However, there were no significant associations of mortality at 1 year with either time to presentation or the door-to-needle time, probably because of our small study sample.

Meta-analysis has shown primary PCI to be superior to thrombolysis in the treatment of STEMI and to benefit long term survival and reduce strokes, recurrent ischemia, and reinfarction [18]. Unfortunately, only a minority of Sri Lankan patients have the opportunity to undergo primary PCI at present. Only 27% of patients from the current study went onto have a cardiac stress test, only 12.3% underwent coronary angiography, and only 7.3% underwent revascularization within 1 year of STEMI. Regionally, in India 7.5–12% of patients presenting with ACS undergo primary PCI and 20% undergo angiography [9, 19]. By comparison, western countries report rates of 56.3% for angiography, 40.4% for percutaneous intervention, and 3.4% for coronary artery bypass grafting for patients presenting with acute STEMI [20, 21]. Cardiac care infrastructure, personnel, and protocols in Sri Lanka need to keep pace with expanding facilities globally to confer the benefits of new developments to patients.

### Limitations

Limitations of this study were its relatively small sample size, that it was a single-center investigation, and that it included only patients in the acute-care setting, which may have led to underestimation of the event rates, since patients who were dead on admission would not have been included in our analysis. Absence of the causes of death for those who died during the first year after STEMI is another notable limitation.

## Conclusions

Sri Lanka still relies heavily on thrombolysis for the acute management of STEMI. Pre-hospital delays remain a critical factor in underutilization of thrombolysis, and recommended door-to-needle time is achieved in only a minority. Primary PCI and use of coronary intervention in the post-infarction period remain underutilized. In an environment that still relies heavily on thrombolysis as the primary treatment option for STEMI, more streamlined services, strict adherence to guidelines, and training of first-contact physicians is paramount in reducing mortality from myocardial infarction.

## References

[1] Wijewardene K, Mohideen MR, Mendis S, Fernando DS, Kulathilaka T, Weerasekara D (2005). Prevalence of hypertension, diabetes and obesity: baseline findings of a population based survey in four provinces in Sri Lanka. Ceylon Med J.

[2] Abeywardena MY (2003). Dietary fats, carbohydrates and vascular disease: Sri Lankan perspectives. Atherosclerosis.

[3] Medical statistics Unit Ministry of Health (2012). Annual Health Bulletin 2012.

[4] Mendis S, Abegunde D, Yusuf S, Ebrahim S, Shaper G, Ghannem H (2005). WHO study on Prevention of REcurrences of Myocardial Infarction and StrokE (WHO-PREMISE). Bull World Health Organ.

[5] Investigators A (2011). Management of acute coronary syndromes in developing countries: acute coronary events-a multinational survey of current management strategies. Am Heart J.

[6] Eagle KA, Goodman SG, Avezum A, Budaj A, Sullivan CM, Lopez-Sendon J (2002). Practice variation and missed opportunities for reperfusion in ST-segment-elevation myocardial infarction: findings from the Global Registry of Acute Coronary Events (GRACE). Lancet.

[7] Fox KA, Goodman SG, Anderson FA, Granger CB, Moscucci M, Flather MD (2003). From guidelines to clinical practice: the impact of hospital and geographical characteristics on temporal trends in the management of acute coronary syndromes. The Global Registry of Acute Coronary Events (GRACE). Eur Heart J.

[8] Rajapakse S, Rodrigo PC, Selvachandran J (2010). Management of acute coronary syndrome in a tertiary care general medical unit in Sri Lanka: how closely do we follow the guidelines?. J Clin Pharm Ther.

[9] Mohanan PP, Mathew R, Harikrishnan S, Krishnan MN, Zachariah G, Joseph J (2013). Presentation, management, and outcomes of 25 748 acute coronary syndrome admissions in Kerala, India: results from the Kerala ACS Registry. Eur Heart J.

[10] Katulanda P, Ranasinghe P, Jayawardena R, Constantine GR, Rezvi Sheriff MH, Matthews DR (2014). The prevalence, predictors and associations of hypertension in Sri Lanka: a cross-sectional population based national survey. Clin Exp Hypertens.

[11] Katulanda P, Constantine GR, Mahesh JG, Sheriff R, Seneviratne RD, Wijeratne S (2008). Prevalence and projections of diabetes and pre-diabetes in adults in Sri Lanka–Sri Lanka Diabetes, Cardiovascular Study (SLDCS). Diabet Med.

[12] Constantine GR, Thenabadu PN (1998). Time delay to thrombolytic therapy–a Sri Lankan perspective. Postgrad Med J.

[13] Constantine GR, Herath JI, Chang AA, Suganthan P, Hewamane BS, Thenabadu PN (1999). Management of acute myocardial infarction in general medical wards in Sri Lanka. Postgrad Med J.

[14] Loch A, Lwin T, Zakaria IM, Abidin IZ, Wan Ahmad WA, Hautmann O (2013). Failure to improve door-to-needle time by switching to emergency physician-initiated thrombolysis for ST elevation myocardial infarction. Postgrad Med J.

[15] World Health Organization (2011). Global status report on noncommunicable diseases 2010.

[16] Chen ZM, Pan HC, Chen YP, Peto R, Collins R, Jiang LX (2005). Early intravenous then oral metoprolol in 45,852 patients with acute myocardial infarction: randomised placebo-controlled trial. Lancet.

[17] Sikri N, Bardia A (2007). A history of streptokinase use in acute myocardial infarction. Tex Heart Inst J.

[18] Keeley EC, Boura JA, Grines CL (2003). Primary angioplasty versus intravenous thrombolytic therapy for acute myocardial infarction: a quantitative review of 23 randomised trials. Lancet.

[19] Xavier D, Pais P, Devereaux PJ, Xie C, Prabhakaran D, Reddy KS (2008). Treatment and outcomes of acute coronary syndromes in India (CREATE): a prospective analysis of registry data. Lancet.

[20] Hasdai D, Behar S, Wallentin L, Danchin N, Gitt AK, Boersma E (2002). A prospective survey of the characteristics, treatments and outcomes of patients with acute coronary syndromes in Europe and the Mediterranean basin; the Euro Heart Survey of Acute Coronary Syndromes (Euro Heart Survey ACS). Eur Heart J.

[21] Fox KA, Goodman SG, Klein W, Brieger D, Steg PG, Dabbous O (2002). Management of acute coronary syndromes. Variations in practice and outcome; findings from the Global Registry of Acute Coronary Events (GRACE). Eur Heart J.

[CR22] The pre-publication history for this paper can be accessed here:http://www.biomedcentral.com/1471-2261/15/1/prepub

